# Highly selective hydrogenation of CO_2_ into C_2+_ alcohols by homogeneous catalysis†
†Electronic supplementary information (ESI) available: Supplementary figures and tables. See DOI: 10.1039/c5sc02000j


**DOI:** 10.1039/c5sc02000j

**Published:** 2015-07-10

**Authors:** Qingli Qian, Meng Cui, Zhenhong He, Congyi Wu, Qinggong Zhu, Zhaofu Zhang, Jun Ma, Guanying Yang, Jingjing Zhang, Buxing Han

**Affiliations:** a Beijing National Laboratory for Molecular Sciences , CAS Key Laboratory of Colloid, Interface and Chemical Thermodynamics , Institute of Chemistry , Chinese Academy of Sciences , Beijing 100190 , China . Email: qianql@iccas.ac.cn ; Email: hanbx@iccas.ac.cn ; Tel: +86-10-62562821

## Abstract

The hydrogenation of CO_2_ to produce alcohols with two or more carbons (C_2+_ alcohols) is of great importance, but is challenging. In this work, we found that a Ru_3_(CO)_12_/Rh_2_(CO)_4_Cl_2_–LiI system could catalyze the reaction effectively in 1,3-dimethyl-2-imidazolidinone (DMI) under mild conditions. Methanol, ethanol, propanol, 2-methyl propanol, butanol, and 2-methyl butanol were produced in the homogeneous catalytic reaction. The C_2+_ alcohols could be generated at 160 °C, which is the lowest temperature reported so far for producing C_2+_ alcohols *via* CO_2_ hydrogenation. The selectivity for the C_2+_ alcohols could be as high as 96.4% at the optimized conditions, which is higher than those reported in the literature. In addition, the catalytic system could be easily recycled. The route of the reaction for forming the C_2+_ alcohols was discussed on the basis of control experiments.

## Abstract 2

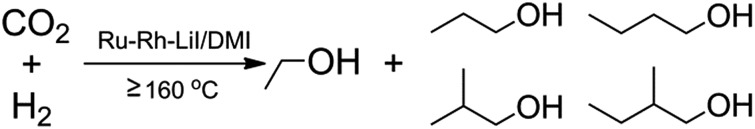
Methanol, ethanol, propanol, 2-methyl propanol, butanol, and 2-methyl butanol were produced in homogeneous CO_2_ hydrogenation with a selectivity for C_2+_ alcohols of 96.4%.

## Introduction

Carbon dioxide (CO_2_) is a renewable, abundant, and cheap C_1_ feedstock.^1^ The use of CO_2_ as a carbon source to produce fuels and value-added chemicals is of great importance for the sustainable development of our society. The transformation of CO_2_ into various compounds, such as urea, salicylic acid, carbonates, polymers, alcohols, and formic acid, has been studied extensively.^2^

The hydrogenation of CO_2_ to produce alcohols is one of the most important routes for converting CO_2_, which has received much attention, especially for producing methanol.^3^ In many cases, ethanol and larger alcohols (C_2+_ alcohols hereafter) are more desirable products, as neat fuels, fuel additives, and chemicals, than methanol.^4^ However, producing C_2+_ alcohols by CO_2_ hydrogenation is more difficult than producing methanol. Up to now, heterogeneous catalysts have been designed and used in the synthesis of C_2+_ alcohols by the catalytic hydrogenation of CO_2_, and some excellent results have been obtained.^5^–^15^ For example, it was found that alkali-promoted Mo/SiO_2_ catalysts could catalyze CO_2_ hydrogenation to form C_2+_ alcohols at 250 °C. The content of C_2+_ alcohols in the alcohol mixture could be 75.6%.^5^ Supported Rh, Fe-based, and Cu-based catalysts were combined for the synthesis of C_2+_ alcohols, and the multi-functional heterogeneous catalysts could promote the reaction effectively at 330–370 °C. The major C_2+_ alcohol was ethanol and the highest selectivity of C_2+_ alcohols in the alcohols was about 70%.^6^ Kurakata *et al.*^7^ reported that [Rh_10_Se]/TiO_2_ could promote the hydrogenation of CO_2_ to produce ethanol at temperatures from 250 to 450 °C, and the highest ethanol selectivity was 83%. Nieskens *et al.*^8^ fabricated a CoMoS based catalyst for synthesizing C_1_–C_3_ alcohols *via* CO_2_ hydrogenation at 340 °C, and the highest C_2+_ alcohol content in the alcohol mixture was 35.6%. Li *et al.*^9^ prepared a K/Cu–Zn–Fe catalyst, which was used in the reaction at 300 °C. The selectivity to C_2+_ alcohols reached 87.1%. Tominaga *et al.*^16^ reported CO_2_ hydrogenation using a Ru–Co homogeneous catalyst at 200 °C, and only methanol and ethanol were formed, with an ethanol selectivity of 26.4% in the alcohol products.

**Scheme 1:**
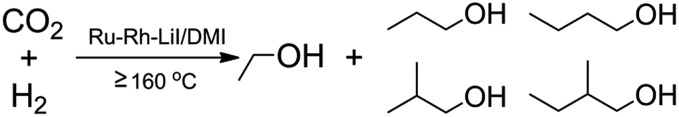
Synthesis of C_2+_ alcohols from CO_2_ hydrogenation.

As discussed above, the synthesis of C_2+_ alcohols by the hydrogenation of CO_2_ has received considerable attention. However, in general, the heterogeneous catalysts suffer from low activity, low C_2+_ alcohol selectivity, and high reaction temperature. There is no doubt that exploration of the routes for highly selective CO_2_ hydrogenation to produce C_2+_ alcohols under relatively mild conditions is of great importance. In this work, we studied the hydrogenation of CO_2_ into C_1–5_ alcohols catalyzed by a Ru–Rh bimetallic homogeneous catalyst using LiI as the promoter (Scheme 1).

It was found that the catalytic system could catalyze the reaction effectively under mild conditions. The liquid products were mainly methanol, ethanol, propanol, 2-methyl propanol, butanol and 2-methyl butanol, including both linear and branched alcohols. The products were distinct from those generated *via* homogeneous CO_2_ or CO hydrogenation reported in the literature. The alcohols could be generated at 160 °C. The selectivity for C_2+_ alcohols could be as high as 96.4% at the optimized conditions. In addition, the catalytic system could be recycled and reused.

## Results and discussion

Different catalytic systems were tested for the reaction, and the results are listed in Table 1. The corresponding selectivities for the different alcohols are shown in detail in Table S1.† When LiI was used as the promoter and 1,3-dimethyl-2-imidazolidinone (DMI) as the solvent, the combined catalyst Ru_3_(CO)_12_/Rh_2_(CO)_4_Cl_2_ could catalyze the hydrogenation of CO_2_ very effectively, with methanol, ethanol, propanol, 2-methyl propanol, butanol, and 2-methyl butanol as the products, and other products being negligible in the reaction solution (Fig. S1†). The alcohol products, including both linear and branched C_1_–C_5_ alcohols, are different from those obtained *via* CO hydrogenation using Ru and/or Rh catalysts, since they give largely linear hydrocarbons over heterogeneous catalysts, and produce only C_1_ and C_2_ oxygenates in homogeneous catalysis.^17^ Very interestingly, the selectivity for C_2+_ alcohols could be as high as 96.4% (Entry 1), which is much higher than those reported in the literature.

**Table 1:** The performances of various catalytic systems for CO_2_ hydrogenation to C_2+_ alcohols^*a*^

Entry	Catalyst	Promoter	Solvent	STY^*c*^ of alcohols	C_2+_OH%
1	Ru_3_(CO)_12_, Rh_2_(CO)_4_Cl_2_	LiI	DMI	12.86	96.4
2^*b*^	Ru_3_(CO)_12_, Rh_2_(CO)_4_Cl_2_	—	DMI	0.36	2.8
3	Ru_3_(CO)_12_, Rh_2_(CO)_4_Cl_2_	KI	DMI	14.36	8.3
4	Ru_3_(CO)_12_, Rh_2_(CO)_4_Cl_2_	LiCl	DMI	16.17	17.7
5	Ru_3_(CO)_12_	LiI	DMI	2.43	0.4
6^*b*^	Rh_2_(CO)_4_Cl_2_	LiI	DMI	1.07	2.9
7	Ru_3_(CO)_12_, Rh_2_(CO)_4_Cl_2_	LiI	NMP	5.11	72.4
8^*b*^	Ru_3_(CO)_12_, Rh_2_(CO)_4_Cl_2_	LiI	1-Methyl piperidine	2.07	0.0
9^*b*^	Ru_3_(CO)_12_, Rh_2_(CO)_4_Cl_2_	LiI	DMF	7.64	0.0
10^*b*^	Ru_3_(CO)_12_, Rh_2_(CO)_4_Cl_2_	LiI	THF	0.0	—
11^*b*^	Ru_3_(CO)_12_, Rh_2_(CO)_4_Cl_2_	LiI	Cyclohexane	0.0	—
12^*b*^	Ru_3_(CO)_12_, Rh_2_(CO)_4_Cl_2_	LiI	Water	1.45	6.5
13^*b*^	RuCl_3_·3H_2_O, Rh_2_(CO)_4_Cl_2_	LiI	DMI	2.73	7.4
14^*b*^	Ru_3_(CO)_12_, RhCl_3_·*x*H_2_O	LiI	DMI	3.38	5.7
15^*b*^	Ru_3_(CO)_12_, Rh_6_(CO)_16_	LiI	DMI	3.40	25.4

^*a*^Reaction conditions: 28.2 μmol Ru catalyst and 51.5 μmol Rh catalyst (based on the metal), 2.26 mmol promoter, 2 mL solvent, 4 MPa CO_2_ and 4 MPa H_2_ (at room temperature), 200 °C and 12 h.

^*b*^Precipitate was observed after the reaction.

^*c*^STY stands for space time yield (C mmol L^–1^ h^–1^), which is one of the commonly used units, especially when multi-metals are utilized.

The promoter LiI played an important role in accelerating the reaction. Without the promoter, a little amount of methanol was generated, and the amount of the C_2+_ alcohols was negligible (Entry 2). In the presence of LiI, the reaction solution was clear after reaction, but black fine metal powder was found when LiI was not added, indicating that LiI could stabilize the catalyst. When LiI was replaced by KI, the catalyst was also stable at the reaction conditions with a high yield of methanol, but the selectivity to C_2+_ alcohols was very low (Entry 3). The results show that the promoter affected the activity, selectivity, and stability of the catalyst. The superiority of LiI in promoting the synthesis of C_2+_ alcohols may be partly attributed to the stronger Lewis acidity of the lithium cation, which could offer suitable coordination sites during the catalytic reaction. The anionic counterpart of the promoter also evidently influenced the catalytic performance. When LiCl was used, the selectivity for C_2+_ alcohols was much lower (Entry 4). The better performance of the iodide anion may be ascribed to its stronger nucleophilicity, which would promote the chain growth reaction.

We also used Ru_3_(CO)_12_ (Entry 5) and Rh_2_(CO)_4_Cl_2_ (Entry 6) separately, but the yield of the product and the selectivity to the C_2+_ alcohols was very low, indicating the synergistic effect of the two catalysts in accelerating the reaction. Thus we choose the space time yield (STY) to express the catalytic activity, which may give an integrated evaluation of the bimetallic catalytic system. The reaction was also carried out in other solvents, and it was demonstrated that DMI was the best solvent for the reaction (Entries 1 and 7–12). One of the main reasons is that the catalyst was stable in DMI, but it was not stable in most of the other solvents used. In *N*-methyl-2-pyrrolidone (NMP), the catalyst was also stable, but the efficiency of the reaction was lower than that in DMI. This indicates that the solvent effect is also important for the reaction. Using LiI as the promoter and DMI as the solvent, the performance of other mixed catalysts, such as RuCl_3_·3H_2_O/Rh_2_(CO)_4_Cl_2_, Ru_3_(CO)_12_/RhCl_3_·*x*H_2_O, and Ru_3_(CO)_12_/Rh_6_(CO)_16_, were also studied (Entries 13–15), but the efficiencies were lower than that of Ru_3_(CO)_12_/Rh_2_(CO)_4_Cl_2_ because of their poor stability. The results above indicate that the catalytic system composed of Ru_3_(CO)_12_/Rh_2_(CO)_4_Cl_2_, LiI, and DMI had good activity, selectivity, and stability for the hydrogenation of CO_2_ to generate C_2+_ alcohols. Therefore, the effects of the reaction conditions were further studied using this catalytic system.

**Fig. 1:**
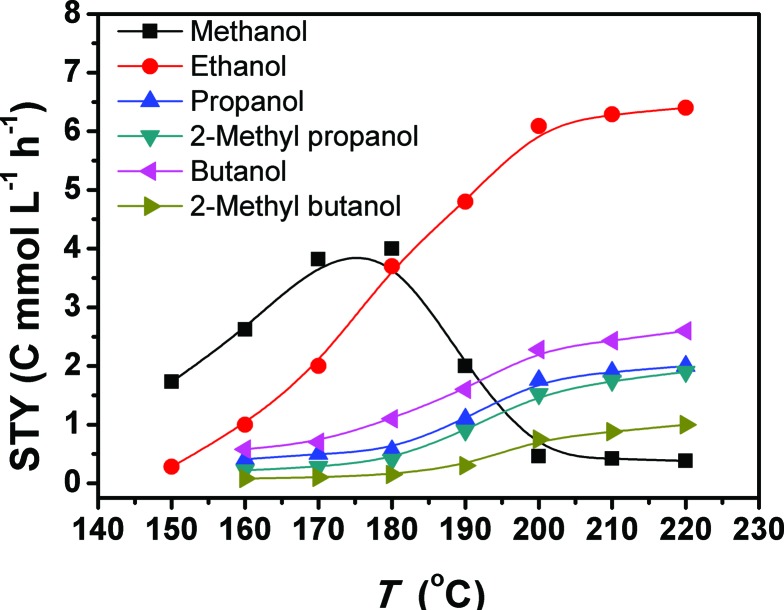
The space time yields (STY) of the alcohols at different temperatures. Reaction conditions: 28.2 μmol Ru_3_(CO)_12_ and 51.5 μmol Rh_2_(CO)_4_Cl_2_ (based on the metal), 2.26 mmol LiI, 2 mL DMI, 4 MPa CO_2_ and 4 MPa H_2_ (at room temperature), and 12 h.

Fig. 1 depicts the results of the CO_2_ hydrogenation conducted at different temperatures. At 150 °C, only methanol and ethanol were formed, and methanol was the major product. When the temperature reached 160 °C, the yields of methanol and ethanol increased, and C_3+_ alcohols emerged. Thus, 160 °C was the initial temperature for the obvious formation of C_3+_ alcohols. So far this is the lowest temperature reported for the formation of these alcohols. The yields of all the alcohols increased as the temperature rose. From 180 to 200 °C, the methanol yield underwent a dramatic drop, accompanied with an evident increase in the yields of the target C_2+_ alcohols. In the range of 200–220 °C, the yields of methanol and ethanol were nearly unchanged with increasing temperature, but the yields of the other alcohols increased continuously with increasing temperature. The main reason is that the methanol formed can be further transformed into ethanol, and the ethanol can be converted into larger alcohols, which will be discussed in more detail in the following paragraphs. The yield of methanol is much lower than that of ethanol in this temperature range because methanol is more reactive than ethanol. Therefore, the methanol generated was converted into ethanol quickly.

The results in Fig. 1 suggest that 200 °C is a suitable temperature. We further studied the effects of other parameters on the reaction at this temperature, and the results are given in Table 2. The corresponding selectivities to different alcohols are shown in Table S2.† The C_2_–C_5_ alcohols were generated at all the conditions. At a fixed pressure ratio of CO_2_ and H_2_ (1 : 1), the total yield of the alcohols and the selectivity to the C_2+_ alcohols increased remarkably as the total pressure was raised from 2 MPa to 10 MPa (Entries 1–5). At the same pressure, the total yield of the alcohols increased with the partial pressure of H_2_ (Entries 4, 6, 7), but the selectivity to C_2+_ alcohols was highest at a CO_2_ : H_2_ pressure ratio of 1 : 1. The dosage of LiI was crucial for the alcohols generation (Entry 2 of Table 1, Entries 4, 8, and 9 of Table 2). When the LiI dosage was in the range of 0–1.13 mmol, both the total yield of the alcohols and the selectivity to C_2+_ alcohols increased significantly with the increase in the dosage. As the LiI dosage was increased to 2.26 mmol, the amount of the alcohols generated decreased slightly but the C_2+_ alcohols selectivity increased greatly. However, as the dosage further increased to 3.39 mmol, the selectivity to C_2+_ alcohols remained high, but the total yield of the alcohols reduced considerably. The main reason may be that more active sites were occupied by iodide anions as an excess amount of LiI was used, inhibiting the hydrogenation reaction. The atomic ratio of Ru and Rh also affected the yield of the reaction. At the same total amount of Ru and Rh (79.7 μmol), 28.2 μmol Ru and 51.5 μmol Rh gave the highest total yield of the alcohols and the selectivity for C_2+_ alcohols (Entries 5 and 6 of Table 1, Entries 4, 10, 11, 12 of Table 2). As expected, the total yield of the alcohols increased with an increasing amount of the catalyst (Entries 4, 13, 14, 15), but the yield was less sensitive to the amount of catalyst as the amount was large enough.

**Table 2:** Effect of reaction parameters on hydrogenation of CO_2_ to alcohols^*a*^

Entry	Ru/Rh [μmol]	LiI [mmol]	CO_2_/H_2_ [MPa]	STY of alcohols	C_2+_ [%]
1	28.2/51.5	2.26	1/1	1.13	77.0
2	28.2/51.5	2.26	2/2	3.39	90.6
3	28.2/51.5	2.26	3/3	5.37	92.6
4	28.2/51.5	2.26	4/4	12.86	96.4
5	28.2/51.5	2.26	5/5	14.10	96.1
6	28.2/51.5	2.26	2/6	20.66	39.0
7	28.2/51.5	2.26	6/2	3.17	84.2
8	28.2/51.5	1.13	4/4	14.25	40.6
9	28.2/51.5	3.39	4/4	5.88	97.1
10	8.0/71.7	2.26	4/4	3.32	84.0
11	39.9/39.9	2.26	4/4	12.07	76.9
12	55.8/23.9	2.26	4/4	8.57	80.4
13	0/0	2.26	4/4	0	—
14	14.1/25.8	2.26	4/4	4.48	47.8
15	42.3/77.3	2.26	4/4	16.31	93.9

^*a*^Reaction conditions: Ru_3_(CO)_12_/Rh_2_(CO)_4_Cl_2_ were used as the catalysts and their dosage was based on the metal, LiI was used as the promoter, 2 mL DMI, 200 °C, and 12 h.

We carried out experiments on the reuse of the catalytic system. After reaction, the alcohols generated in the reaction were removed under vacuum, which was confirmed by GC analysis. Then the catalyst, solvent (DMI), and the LiI were used directly for the next run. The results of the reuse experiments are given in Table S3.† The yield of the total alcohols and the selectivity to C_2+_ alcohols did not change obviously after five cycles (12 h each cycle), indicating that the catalyst was stable for at least 60 h at this temperature.

**Fig. 2:**
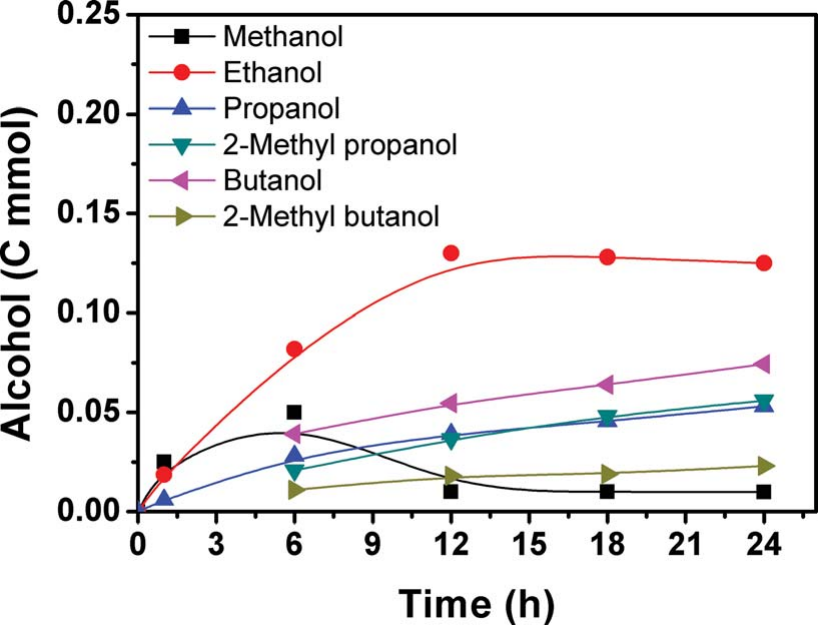
Time course of the alcohol formation. Reaction conditions: 28.2 μmol Ru_3_(CO)_12_ and 51.5 μmol Rh_2_(CO)_4_Cl_2_ (based on the metal), 2.26 mmol LiI, 2 mL DMI, 4 MPa CO_2_ and 4 MPa H_2_ (at room temperature), and 200 °C.

Fig. 2 presents the time course for the formation of the alcohols. Methanol, ethanol and propanol were generated within 1 h and their yields increased with time. After 6 h, a considerable amount of 2-methyl propanol, butanol, and 2-methyl butanol could be detected and their amounts increased with time. The methanol content began to decrease quickly and the amounts of the higher alcohols increased continuously with the reaction proceeding. After 12 h, the methanol content was low and did not change considerably with time. At the same time, the ethanol content began to decrease slowly, and the content of the C_3+_ alcohols continued to increase with reaction time. The yield of methanol passed through a maximum with increasing reaction time. With the increase of reaction time, some of the methanol is transformed into ethanol and the ethanol can be further converted, and so on.

The results above suggest that methanol was formed from CO_2_ and H_2_ in the reaction. The methanol acts as the intermediate for the generation of ethanol, and the ethanol can be converted into larger alcohols in the reaction process. In order to obtain more evidence to support this argument, we carried out tracer experiments by adding small amounts of ^13^CH_3_OH or ^13^C_2_H_5_OH in the reaction system at 200 °C with a reaction time of 12 h. The GC-MS results with ^13^CH_3_OH and ^13^C_2_H_5_OH are shown in Fig. S2 and S3,† respectively. When ^13^CH_3_OH was used as the tracer, C_2+_ alcohols containing ^13^C were yielded. Similarly, when the ^13^C_2_H_5_OH tracer was added in the reaction system, ^13^C was present in some C_3+_ alcohols. Therefore, it can be concluded that in the hydrogenation of CO_2_ for obtaining the alcohols, the methanol and ethanol formed act as intermediates for forming the larger alcohols.

Only methanol and ethanol were produced as alcohol products in the homogeneous CO_2_ hydrogenation.^16^ It was also reported that only C_1_ and C_2_ oxygenates were yielded *via* CO hydrogenation using homogeneous Ru and/or Rh catalysts.^17^,^18^ Whereas the alcohols generated by our catalytic system included C_1_–C_5_ alcohols of both linear and branched structures. This suggests that the reaction pathway of CO_2_ hydrogenation using our catalytic system is obviously different from those of the CO_2_ or CO hydrogenation reported in the literature.^16^–^18^

## Conclusions

In summary, we have studied the performance of different catalysts, promoters, and solvents for the synthesis of C_2+_ alcohols by the hydrogenation of CO_2_. It is discovered that Ru_3_(CO)_12_, Rh_2_(CO)_4_Cl_2_, and LiI exhibit an excellent synergistic effect in catalyzing the reaction using DMI as the solvent. The Ru_3_(CO)_12_/Rh_2_(CO)_4_Cl_2_/LiI-DMI homogeneous catalytic system can catalyze the reaction effectively and selectively at relatively mild conditions. The target C_2+_ alcohols start to form at 160 °C. The selectivity to the C_2+_ alcohols can reach 96.4%, and the catalytic system can be reused. In the reaction, methanol is first formed, and the small alcohol can act as the intermediate for generating the larger ones. The C_2+_ alcohols include both linear and branched alcohols, which is distinct from those produced *via* homogeneous CO_2_ or CO hydrogenation reported in the literature. We believe that many other catalytic systems can be explored for the hydrogenation of CO_2_ by combination of various homogeneous catalysts, co-catalysts, and solvents.

## Experimental

### Chemicals

Ruthenium carbonyl (Ru_3_(CO)_12_, purity > 98%) was purchased from Adamas Reagent, Ltd. Tetracarbonyl-di-μ-chlorodirhodium(i) (Rh_2_(CO)_4_Cl_2_, Rh 50.1–52.9%), rhodium(iii) chloride hydrate (RhCl_3_·*x*H_2_O, Rh 38.5–45.5%), anhydrous lithium iodide (LiI, 99.95%), potassium iodide, (KI, 99.9%), and 1-methyl piperidine (99%) were obtained from Alfa Aesar China Co., Ltd. Hexarhodiumhexadecacarbonyl (Rh_6_(CO)_16_, 98%) was provided by J&K Chemical Ltd. (Shanghai). Ruthenium(iii) chloride hydrate (RuCl_3_·3H_2_O, Ru 36.7%) was provided by Shenyang Jinke Reagent Co., Ltd. 1,3-Dimethyl-2-imidazolidinone (DMI, 99%) was purchased from TCI Shanghai Co., Ltd. *N*-Methyl-2-pyrrolidone (NMP, 99.5%), *N*,*N*-dimethylformamide (DMF, 99.5%) and cyclohexane (99.5%) were provided by Sinopharm Chemical Reagent Co., Ltd. Tetrahydrofuran (THF, A.R. Grade) was obtained from Beijing Chemical Company. Toluene (99.8%, HPLC) was obtained from Xilong Chemical Co., Ltd. Methanol-^13^C (99 atom% ^13^C) and ethanol-^13^C_2_ (99 atom% ^13^C) were purchased from Sigma-Aldrich Co. LLC. The CO_2_ (99.99%) and H_2_ (99.99%) were provided by Beijing Analytical Instrument Company.

### Hydrogenation of CO_2_

All the reactions were conducted in a 16 mL Teflon-lined stainless steel reactor equipped with a magnetic stirrer. In a typical experiment, known amounts of the Ru and/or Rh catalysts, LiI or another promoter, tracer (methanol-^13^C or ethanol-^13^C_2_ if used), and 2 mL solvent were loaded into the reactor. The reactor was sealed and purged three times with CO_2_ of 3 MPa, and then CO_2_ and hydrogen were charged to the desired pressure at room temperature, respectively. The reactor was placed in an air bath of constant temperature, and the magnetic stirrer was started at 800 rpm. After reaction, the reactor was cooled in an ice-water bath for 1 h, the residual gas was released carefully in a hood. The liquid mixture was analyzed by GC (Agilent 7890B) equipped with a flame ionization detector and a HP-5 capillary column (0.32 mm in diameter and 30 m in length) using toluene as the internal standard. Identification of the liquid products was done using a GC-MS (SHIMADZU-QP2010) as well as by comparing the retention times of the standards in the GC traces (Fig. S4†). The yields of the products were calculated from the GC data.

To test the reusability of the catalytic system, the alcohols formed in the reaction were removed at 80 °C under vacuum for 1.5 h, and the catalytic system (Ru_3_(CO)_12_–Rh_2_(CO)_4_Cl_2_–LiI/DMI) was used directly for the next run.

## Supplementary Material

Supplementary information
