# Supplementary material for: Highly selective hydrogenation of CO2 into C2+ alcohols by homogeneous catalysis
Source: Chem Sci. 2015 Jul 10;6(10):5685–9. doi: 10.1039/c5sc02000j (PMC5947507; doi:10.1039/c5sc02000j)
Supplement: Supplementary file 1 [file SC-006-C5SC02000J-s001.pdf]

Electronic Supplementary Material (ESI) for Chemical Science.
This journal is © The Royal Society of Chemistry 2015

Supplementary Information

Highly Selective Hydrogenation of CO_2 into C_{2+} Alcohols by Homogeneous Catalysis

Qingli Qian*, Meng Cui, Zhenhong He, Congyi Wu, Qinggong Zhu, Zhaofu Zhang, Jun Ma, Guanying Yang, Jingjing Zhang and Buxing Han*

Beijing National Laboratory for Molecular Sciences, CAS Key Laboratory of Colloid, Interface and Chemical Thermodynamics, Institute of Chemistry, Chinese Academy of Sciences, Beijing 100190, China.

Supplementary Figures

Chromatogram (TIC)

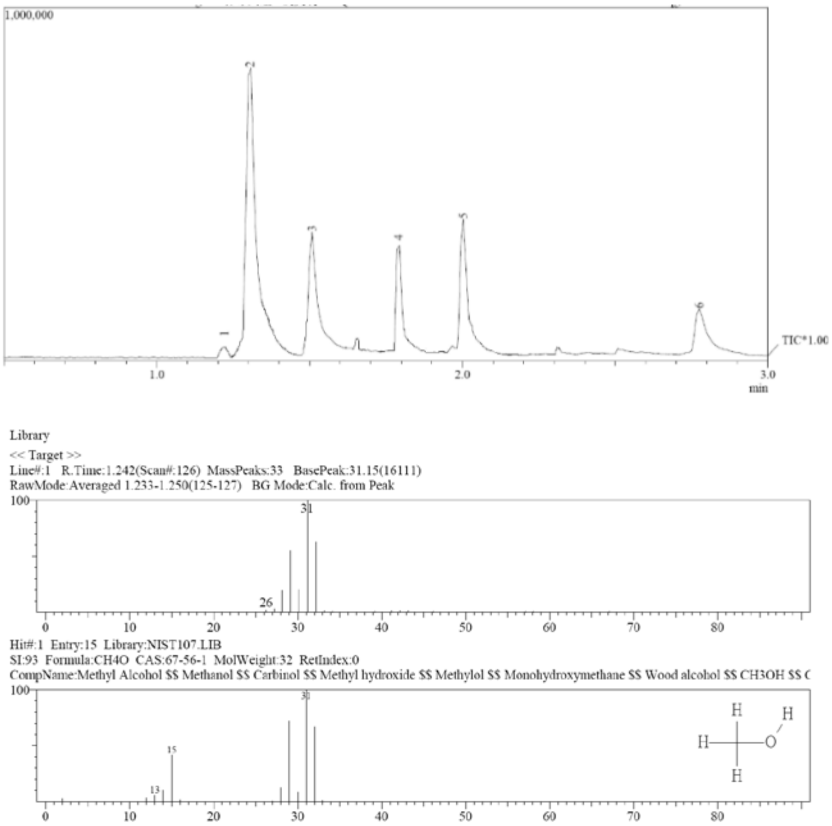

<< Target >>
Line#:2 R.Time:1.308(Scan#:134) MassPeaks:44 BasePeak:31.15(209859)
RawMode:Averaged 1.300-1.317(133-135) BG Mode:Calc. from Peak

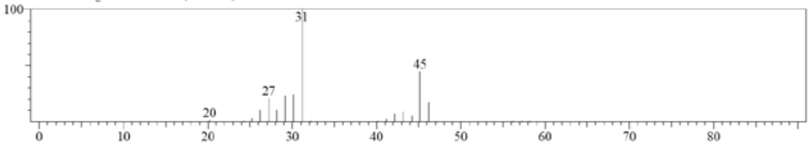

Hit#:1 Entry:51 Library:NIST107.LIB
SI:92 Formula:C2H6O CAS:64-17-5 MolWeight:46 RetIndex:0
CompName:Ethanol $$ Ethyl alcohol $$ Alcohol $$ Alcohol anhydrous $$ Algrain $$ Anhydrol $$ Denatured ethanol $$ Ethyl hydrate $$ Ethyl hyd

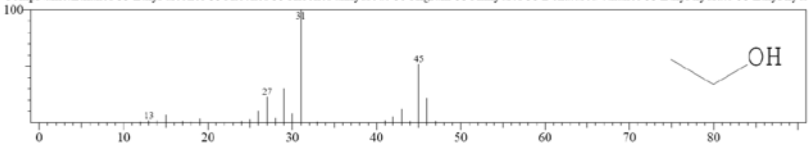

<< Target >>
Line#:3 R.Time:1.508(Scan#:158) MassPeaks:35 BasePeak:31.20(118710)
RawMode:Averaged 1.500-1.517(157-159) BG Mode:Calc. from Peak

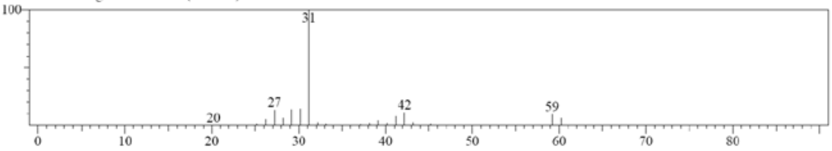

Hit#:1 Entry:144 Library:NIST08.LIB
SI:96 Formula:C3H8O CAS:71-23-8 MolWeight:60 RetIndex:562
CompName:1-Propanol $$ Propyl alcohol $$ n-Propan-1-ol $$ n-Propanol $$ n-Propyl alcohol $$ Ethylcarbinol $$ Optal $$ Osmosol extra $$ Propa

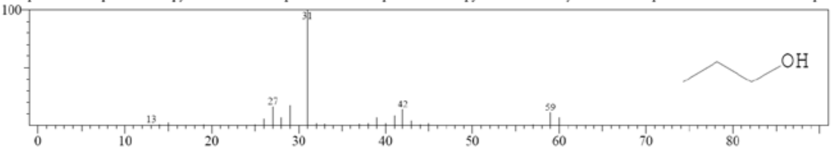

<< Target >>
Line#:4 R.Time:1.783(Scan#:191) MassPeaks:44 BasePeak:43.20(70853)
RawMode:Averaged 1.775-1.792(190-192) BG Mode:Calc. from Peak

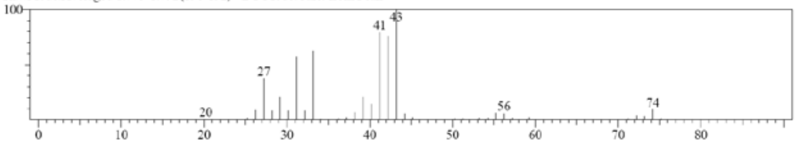

Hit#:1 Entry:468 Library:NIST21.LIB
SI:94 Formula:C4H10O CAS:78-83-1 MolWeight:74 RetIndex:0
CompName:1-Propanol, 2-methyl-

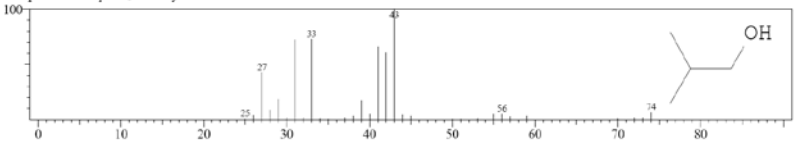

<< Target >>
Line#:5 R.Time:2.008(Scan#:218) MassPeaks:55 BasePeak:31.20(61330)
RawMode:Averaged 2.000-2.017(217-219) BG Mode:Calc. from Peak

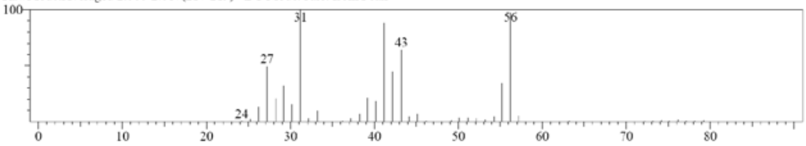

Hit#:1 Entry:396 Library:NIST08.LIB
SI:94 Formula:C4H10O CAS:71-36-3 MolWeight:74 RetIndex:662
CompName:1-Butanol $$ Butyl alcohol $$ n-Butan-1-ol $$ n-Butanol $$ n-Butyl alcohol $$ Butyl hydroxide $$ CCS 203 $$ Hemostyp $$ Methylol

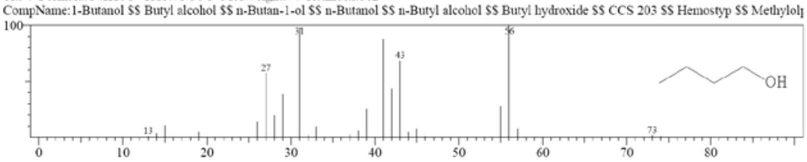

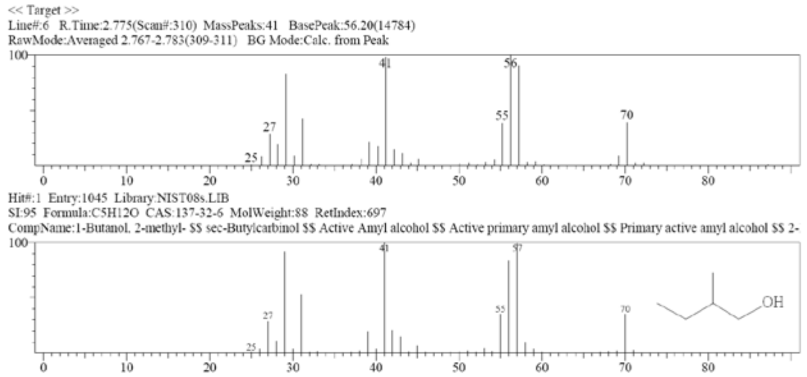

Fig. S1. GC-MS spectra of the product of Entry 1 in Table 1.

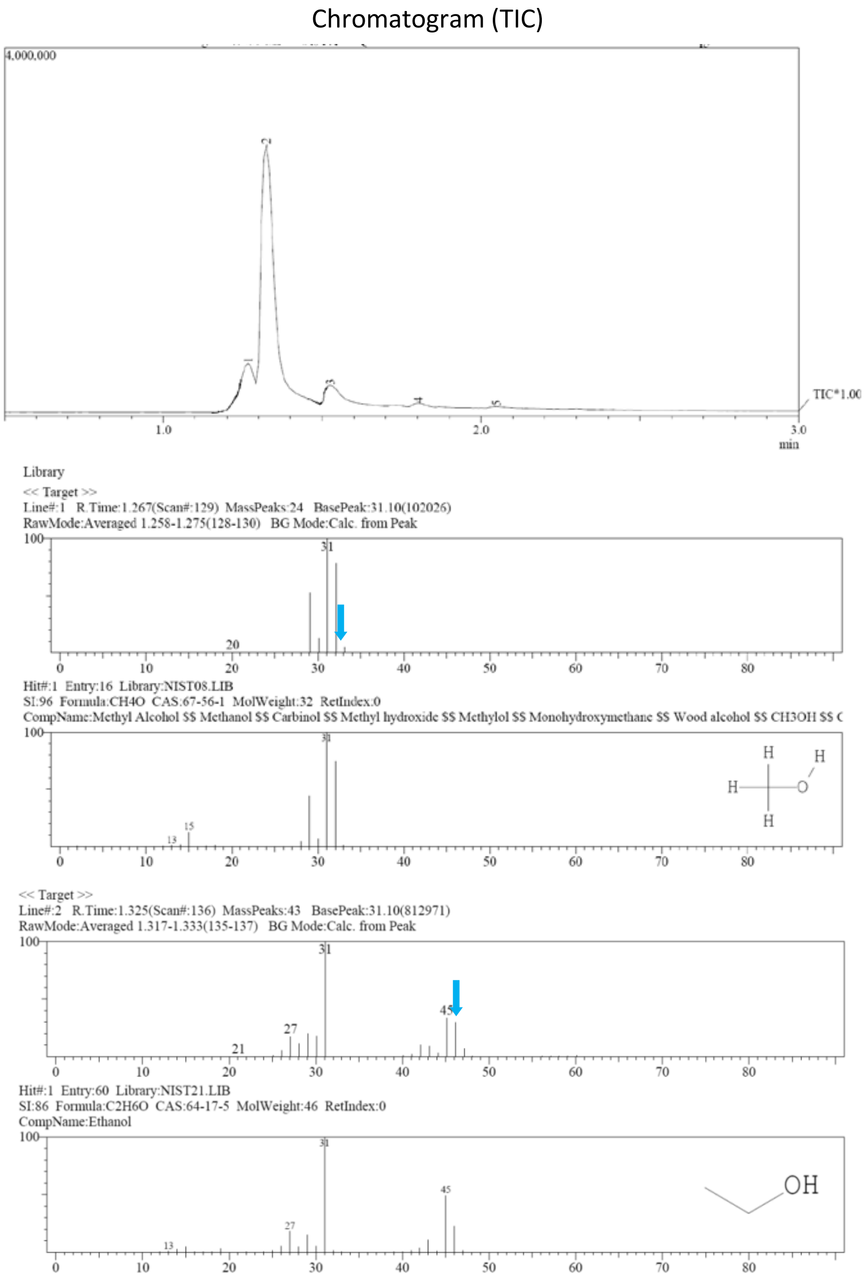

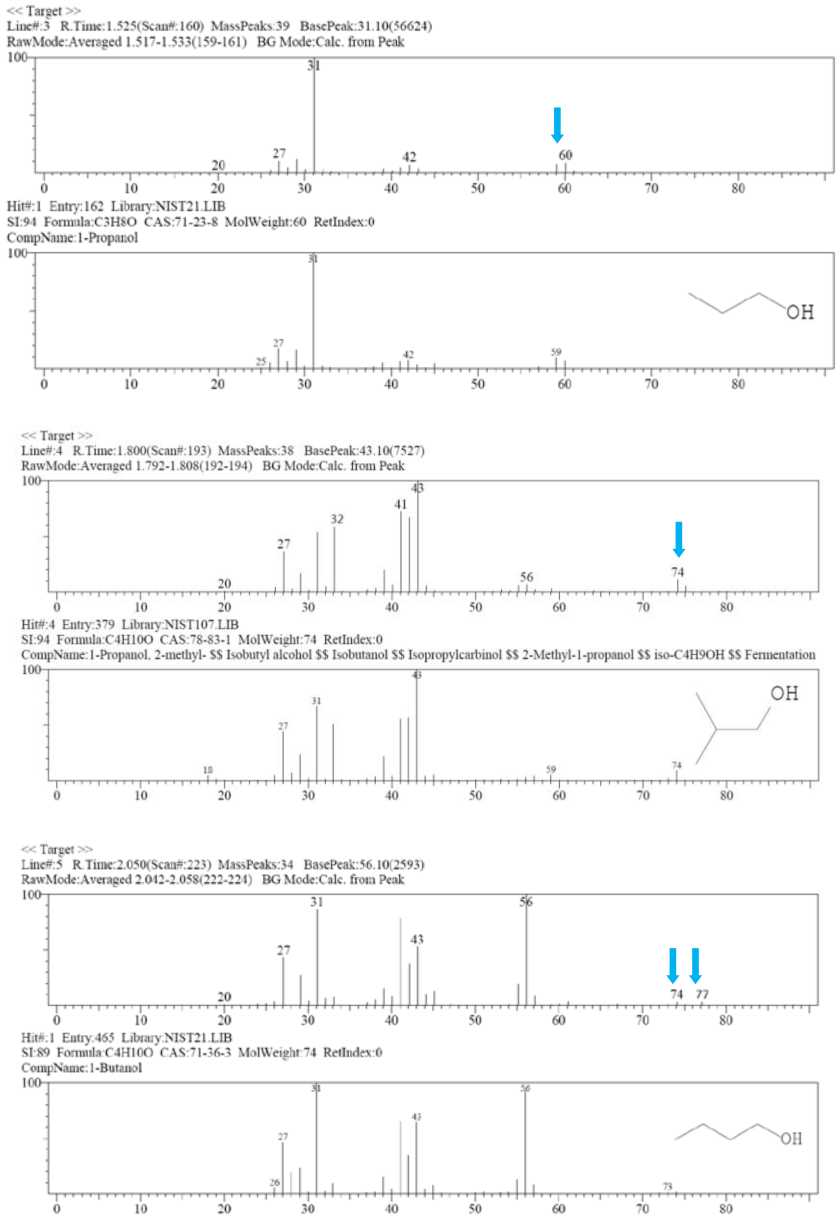

Fig. S2. GC-MS spectra of CH_3OH-^{13}C tracer experiment.

Reaction conditions: 28.2 µmol $Ru_3(CO)_{12}$ and 51.5 µmol $Rh_2(CO)_4Cl_2$ (based on metal), 2.26 mmol LiI, 2 mL DMI, 25 µL methanol-^{13}C (0.62 mmol), 4 MPa CO_2 and 4 MPa H_2 (at room temperature), 200 ^{o}C, 12 hrs.

Chromatogram (TIC)

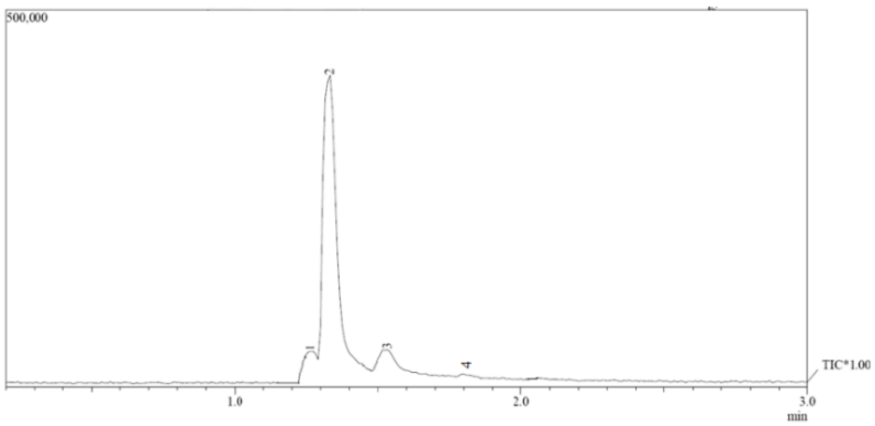

Library
<< Target >>
Line#:1 R.Time:1.258(Scan#:128) MassPeaks:15 BasePeak:31.10(5336)
RawMode:Averaged 1.250-1.267(127-129) BG Mode:Calc. from Peak

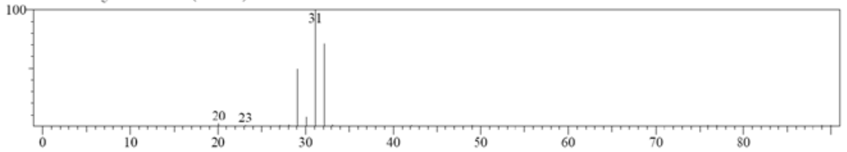

Hit#:1 Entry:15 Library:NIST147.LIB
SI:92 Formula:CH4O CAS:67-56-1 MolWeight:32 RetIndex:0
CompName:Methyl Alcohol $$ Methanol $$ Carbinol $$ Methyl hydroxide $$ Methylol $$ Monohydroxymethane $$ Wood alcohol $$ CH3OH $$ (

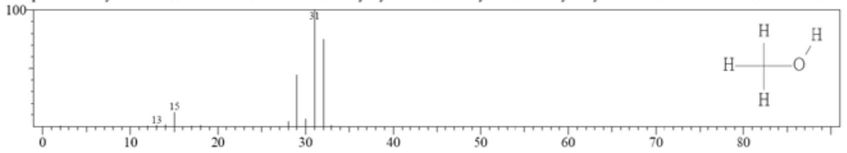

<< Target >>
Line#:2 R.Time:1.333(Scan#:137) MassPeaks:37 BasePeak:32.10(89468)
RawMode:Averaged 1.325-1.342(136-138) BG Mode:Calc. from Peak

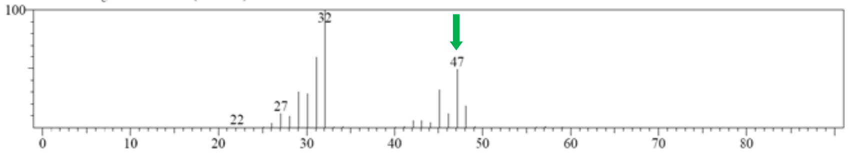

Hit#:1 Entry:60 Library:NIST21.LIB
SI: Formula:C2H6O CAS:64-17-5 MolWeight:46 RetIndex:0
CompName:Ethanol

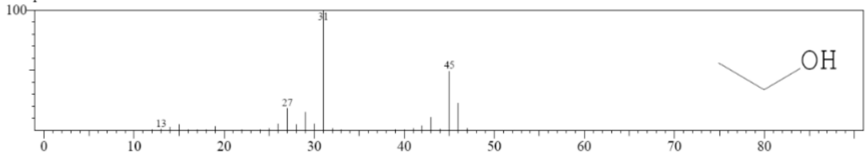

<< Target >>
Line#:3 R.Time:1.533(Scan#:161) MassPeaks:28 BasePeak:31.10(10605)
RawMode:Averaged 1.525-1.542(160-162) BG Mode:Calc. from Peak

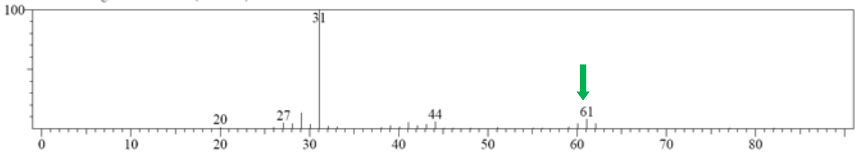

Hit#:1 Entry:150 Library:NIST08s.LIB
SI:87 Formula:C3H8O CAS:71-23-8 MolWeight:60 RetIndex:562
CompName:1-Propanol $$ Propyl alcohol $$ n-Propan-1-ol $$ n-Propanol $$ n-Propyl alcohol $$ Ethylcarbinol $$ Optal $$ Osmosol extra $$ Propa

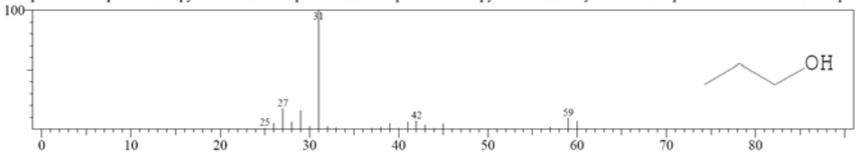

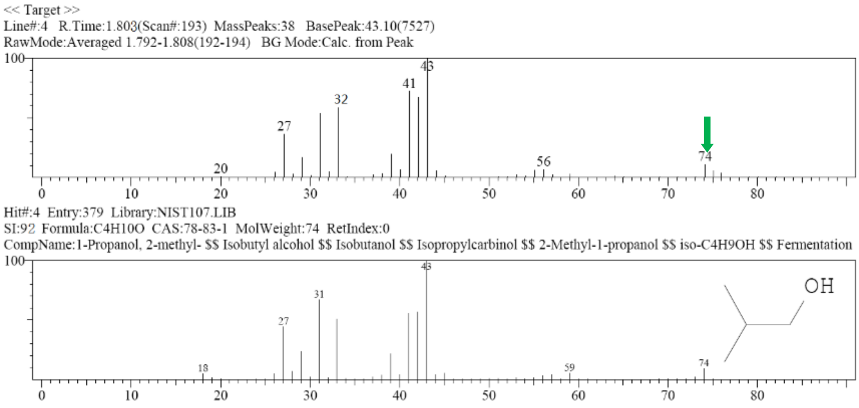

Fig. S3. GC-MS spectra of C_2H_5OH-$^{13}C_2$ tracer experiment

Reaction conditions: 28.2 μmol $Ru_3(CO)_{12}$ and 51.5 μmol $Rh_2(CO)_4Cl_2$ (based on metal), 2.26 mmol LiI, 2 mL DMI, 25 μL ethanol-$^{13}C_2$ (0.43 mmol), 4 MPa CO_2 and 4 MPa H_2 (at room temperature), 200 ^oC, 12 hrs.

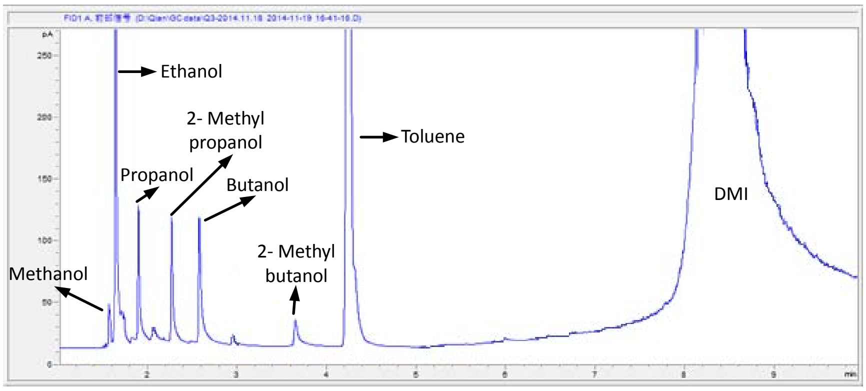

Fig. S4. Representative GC trace of reaction solution after CO_2 hydrogenation with internal standard toluene. The reaction condition is the same as that given in Entry 1 of Table 1.

Table S1. The performances of various catalytic systems for CO_2 hydrogenation to C_{2+} alcohols (with every alcohol).[a]

Entry	Catalyst	Promoter	Solvent	STY (C-mmol/L·h)							C_{2+}alcohol selectivity, %
				Methanol	Ethanol	Propanol	2-methyl propanol	Butanol	2-methyl butanol	Total	
1	$Ru_3(CO)_{12}$/ $Rh_2(CO)_4Cl_2$	LiI	DMI	0.46	6.09	1.76	1.52	2.28	0.75	12.86	96.4
2[b]	$Ru_3(CO)_{12}$/ $Rh_2(CO)_4Cl_2$	-	DMI	0.35	0.01	-	-	-	-	0.36	2.8
3	$Ru_3(CO)_{12}$, $Rh_2(CO)_4Cl_2$	KI	DMI	13.17	0.90	0.11	0.09	0.06	0.03	14.36	8.3
4	$Ru_3(CO)_{12}$, $Rh_2(CO)_4Cl_2$	LiCl	DMI	13.3	2.43	0.34	0.10	-	-	16.17	17.7
5	$Ru_3(CO)_{12}$	LiI	DMI	2.42	0.01	-	-	-	-	2.43	0.4
6[b]	$Rh_2(CO)_4Cl_2$	LiI	DMI	1.04	0.03	-	-	-	-	1.07	2.9
7	$Ru_3(CO)_{12}$, $Rh_2(CO)_4Cl_2$	LiI	NMP	1.41	1.07	0.49	0.68	1.19	0.27	5.11	72.4
8[b]	$Ru_3(CO)_{12}$, $Rh_2(CO)_4Cl_2$	LiI	1-Methyl piperidine	2.07	-	-	-	-	-	2.07	0.0
9[b]	$Ru_3(CO)_{12}$, $Rh_2(CO)_4Cl_2$	LiI	DMF	7.64	-	-	-	-	-	7.64	0.0
10[b]	$Ru_3(CO)_{12}$, $Rh_2(CO)_4Cl_2$	LiI	THF	-	-	-	-	-	-	-	-
11[b]	$Ru_3(CO)_{12}$, $Rh_2(CO)_4Cl_2$	LiI	Cyclohex ane	-	-	-	-	-	-	-	-
12[b]	$Ru_3(CO)_{12}$, $Rh_2(CO)_4Cl_2$	LiI	Water	1.36	0.09	-	-	-	-	1.45	6.5
13[b]	$RuCl_3·3H_2O$, $Rh_2(CO)_4Cl_2$	LiI	DMI	2.52	0.21	-	-	-	-	2.73	7.4
14[b]	$Ru_3(CO)_{12}$, $RhCl_3·xH_2O$	LiI	DMI	3.20	0.18	-	-	-	-	3.38	5.7
15[b]	$Ru_3(CO)_{12}$, $Rh_6(CO)_{16}$	LiI	DMI	2.54	0.82	0.04	-	-	-	3.40	25.4

[a] Reaction conditions: 28.2 µmol Ru catalyst and 51.5 µmol Rh catalyst (based on the metal), 2.26 mmol promoter, 2 mL solvent, 4 MPa CO_2 and 4 MPa H_2 (at room temperature), 200 ^oC, 12 hrs. STY stands for space time yield.

[b] Precipitate was observed after the reaction.

Table S2. Effect of reaction parameters on hydrogenation of CO_2 to alcohols (with every alcohol).[a]

Entry	Ru/Rh [μmol]	LiI [mmol]	CO_2/H_2 [MPa]	STY [C-mmol/L·h]							$C_{2+}OH$ selectivity, %
				Methanol	Ethanol	Propanol	2-methyl propanol	Butanol	2-methyl butanol	Total	
1	28.2/51.5	2.26	1/1	0.26	0.45	0.14	0.23	0.05	0	1.13	77.0
2	28.2/51.5	2.26	2/2	0.32	1.19	0.41	0.57	0.75	0.15	3.39	90.6
3	28.2/51.5	2.26	3/3	0.40	2.06	0.75	0.86	1.03	0.27	5.37	92.6
4	28.2/51.5	2.26	4/4	0.46	6.09	1.76	1.52	2.28	0.75	12.86	96.4
5	28.2/51.5	2.26	5/5	0.55	8.19	1.65	1.38	1.75	0.58	14.10	96.1
6	28.2/51.5	2.26	2/6	12.61	5.43	0.85	0.45	1.13	0.19	20.66	39.0
7	28.2/51.5	2.26	6/2	0.50	1.09	0.39	0.28	0.68	0.23	3.17	84.2
8	28.2/51.5	1.13	4/4	8.47	2.38	0.90	0.62	1.61	0.27	14.25	40.6
9	28.2/51.5	3.39	4/4	0.17	2.76	0.96	0.77	0.91	0.31	5.88	97.1
10	8.0/71.7	2.26	4/4	0.53	1.46	0.59	0.27	0.42	0.05	3.32	84.0
11	39.9/39.9	2.26	4/4	2.79	5.51	0.94	0.82	1.72	0.29	12.07	76.9
12	55.8/23.9	2.26	4/4	1.68	3.48	0.61	1.05	1.37	0.38	8.57	80.4
13	0/0	2.26	4/4	0	0	0	0	0	0	0	-
14	14.1/25.8	2.26	4/4	2.34	0.95	0.28	0.32	0.48	0.11	4.48	47.8
15	42.3/77.3	2.26	4/4	0.99	7.89	2.20	1.93	2.37	0.93	16.31	93.9

[a] Reaction conditions: $Ru_3(CO)_{12}/Rh_2(CO)_4Cl_2$ was used as catalysts and their dosage was based on metal, LiI was used as promoter, 2 mL DMI, 200 ^oC,12 hrs.

Table S3. The results to test the recyclability of catalyst for CO_2 hydrogenation reaction.[a]

Reaction cycles	STY [C-mmol/L·h]							$C_{2+}OH$ selectivity, %
	Methanol	Ethanol	Propanol	2-methyl propanol	Butanol	2-methyl butanol	Total	
1	0.46	6.09	1.76	1.52	2.28	0.75	12.86	96.4
2	0.43	5.92	1.85	1.48	2.35	0.79	12.82	96.6
3	0.49	6.18	1.69	1.55	2.12	0.67	12.70	96.1
4	0.38	5.99	1.70	1.61	2.31	0.63	12.62	97.0
5	0.47	6.02	1.66	1.58	2.10	0.66	12.49	96.2

[a] Reaction conditions: 28.2 μmol $Ru_3(CO)_{12}$ and 51.5 μmol $Rh_2(CO)_4Cl_2$ (based on metal), 2.26 mmol LiI, 2 mL DMI, 4 MPa CO_2 and 4 MPa H_2 (at room temperature), 200 ^oC, 12 hrs.
